# Self-Care and Quality of Life of Ostomy Patients: A Structural Equation Modeling Analysis

**DOI:** 10.3390/nursrep14040247

**Published:** 2024-11-08

**Authors:** Ilaria Marcomini, Paolo Iovino, Laura Rasero, Duilio Fiorenzo Manara, Ercole Vellone, Giulia Villa

**Affiliations:** 1 Center for Nursing Research and Innovation, Faculty of Medicine and Surgery, Vita-Salute San Raffaele University, 20132 Milan, Italy; marcomini.ilaria@unisr.it (I.M.); manara.duilio@unisr.it (D.F.M.); villa.giulia@unisr.it (G.V.); 2 Department of Health Sciences, University of Florence, 50134 Florence, Italy; l.rasero@unifi.it; 3 Department of Biomedicine and Prevention, University of Rome Tor Vergata, 00133 Rome, Italy; ercole.vellone@uniroma2.it; 4 Department of Nursing and Obstetrics, Faculty of Health Sciences, Wroclaw Medical University, 50-367 Wroclaw, Poland

**Keywords:** quality of life, self-care, self-care maintenance, self-care monitoring, ostomy patients, SEM, nursing

## Abstract

**Background.** Previous research has shown that patients with ostomy frequently exhibit a low health-related quality of life (HRQoL). Self-care is a key element that influences their HRQoL. However, the evidence regarding the relationship between these two constructs in patients with ostomy is still not clear. **Materials and Methods.** This was a secondary analysis of an Italian multicenter, observational, longitudinal study. Participants were recruited from seven outpatient ostomy care clinics in central and northern Italy. The Stoma-Specific Quality of Life Questionnaire (Stoma QoL) and the Ostomy Self-Care Index (OSCI) were administered to the participants. The relationship between self-care and HRQoL was analyzed using structural equation modeling. **Results.** A total of 521 patients were enrolled. Our results emphasized that self-care is a predictor of HRQoL among people with ostomy. Self-care maintenance and self-care monitoring had a positive effect on HRQoL (self-care maintenance: β = 0.506, *p* < 0.001; self-care monitoring: β = 0.303, *p* < 0.001). The model exhibited acceptable fit indices: χ^2^ (151, *n*= 521) = 516.447, *p* ≤ 0.001, comparative fit index (CFI) = 0.97, Tucker–Lewis index (TLI) = 0.96, root mean square error of approximation (RMSEA) = 0.068 (90% CI, 0.062–0.075), *p* < 0.001, and standardized root mean square residual (SRMR) = 0.038. **Conclusions.** The structural equation model tested the causal relationship between self-care and HRQoL in people with a stoma, demonstrating for the first time that inadequate self-care in patients with ostomy may lead to lower HRQoL. Thus, to enhance HRQoL, interventions should be designed to improve self-care behaviors. Future research should investigate potential mediating variables in the relationship between self-care and HRQoL.

## 1. Background

International data report that approximately 2 million people are living with a stoma. Of these, 650,000 are European [1], and 70,000 are Italian. The creation of an ostomy, whether temporary or permanent, often leads to significant changes in a patient’s daily life. These changes include alterations in body image, social interactions, and emotional well-being, as well as practical challenges related to ostomy care and management [2]. People with a stoma often face distressing factors such as fear of leaks, flatulence, and shame regarding the device and its use, which can profoundly compromise well-being and social relationships. The ensuing psychological and social adaptations [3] often result in poor overall quality of life (QoL).

The World Health Organization’s Quality of Life Group (WHOQoL Group) defines QoL as a multidimensional concept that encompasses an individual’s perception of his or her life, taking into account cultural and value systems and their goals, expectations, norms, and concerns [4]. Health-related quality of life (HRQoL) is a comprehensive concept that captures a range of experiences and reflects individuals’ views on both the beneficial and detrimental aspects of their lives, incorporating physical, emotional, social, and cognitive well-being. HRQoL also declines in conjunction with physical discomfort or other disease- and treatment-related symptoms [5]. A structured measurement of HRQoL is considered essential for fostering patient-centered care [6] as this construct also has a highly significant impact on mortality and morbidity [7,8,9].

Previous studies have reported that patients with ostomy frequently have poor HRQoL [10,11,12,13]. This is not surprising, as individuals who have undergone stoma surgery often face limitations in performing activities of daily living, difficulties during sexual intercourse, challenges during social interactions, negative emotions arising from the presence of the stoma, economic difficulties, changes in sleep patterns, reduced physical well-being, and stoma-related complications [11,13,14,15].

Numerous factors can affect HRQoL in individuals with an ostomy. Previous research has indicated that being younger than 60 years old [16], possessing a robust social support network [17], and having effective psychosocial adjustment abilities [18] are associated with better HRQoL. In addition, HRQoL is influenced by personal characteristics and psychological health. This aspect is especially important because symptoms of depression and anxiety tend to worsen HRQoL, whereas coping strategies, such as ego defense mechanisms, tend to improve it [17]. In addition, ostomy placement complications and low self-care capabilities are significant predictors of poor HRQoL [12,17,19,20,21,22].

Self-care constitutes a crucial aspect closely associated with HRQoL. Within the chronic illness context, self-care refers to the process of managing a disease while preserving health through health-promoting behaviors [23]. Riegel’s Middle-Range Theory of Self-Care of Chronic Illness [23] defines self-care as a dynamic process that takes into account various factors influencing individuals with chronic conditions. This theory delineates three essential components: self-care maintenance, self-care monitoring, and self-care management. Self-care maintenance encompasses regular efforts to preserve health, whereas self-care monitoring pertains to identifying and analyzing symptoms. Self-care management involves making educated judgments derived from monitoring outcomes and executing problem-solving solutions. Research has shown that self-care behaviors are positively associated with HRQoL in several chronic conditions including heart failure [24,25], chronic obstructive pulmonary disease [26], and chronic venous disease [27]. In individuals with an ostomy, self-care refers to a decision-making process that includes actions to maintain the physiological stability of the stoma and peristomal skin (self-care maintenance), recognize problems and complications (self-care monitoring), and manage these problems (self-care management) [23,28].

So far, prior studies investigating the relationship between self-care and HRQoL have adopted assessment tools that do not sufficiently capture the multidimensional nature of self-care [12,19,20,21,22]. For example, Ayalon and colleagues [19] focused solely on patients‘ ability to manage bodily waste and personal hygiene, neglecting other critical behaviors such as the recognition and management of potential complications associated with an ostomy. Another group of authors [20] only considered the knowledge, skills, and attitudes related to ostomy care or ostomy and pouch management skills [12,19,21], thereby failing to fully represent the self-care construct in their analyses.

Given the gaps identified in the literature, this study explored the relationship between self-care and HRQoL in people with ostomy. We hypothesized that self-care behaviors influence the HRQoL in patients with ostomy.

## 2. Materials and Methods

### 2.1. Design

This was a secondary analysis of an Italian multicenter, observational, longitudinal study. The data were part of a larger survey aimed at describing the level of self-care among patients with ostomy and their caregivers [29].

### 2.2. Sample and Data Collection

Participants were recruited from seven outpatient clinics specializing in ostomy care across central and northern Italian healthcare settings. Eligibility for recruitment was based on the following inclusion criteria: (a) an ostomy created at least one month prior to enrolment, including elective and urgent cases; (b) an age of over 17 years; (c) proficiency in Italian; and (d) willingness to provide written consent to join the study. Patients with severe cognitive impairment were excluded. Questionnaires were administered in paper form. Patients who met the inclusion criteria completed the questionnaires independently in the outpatient clinics.

### 2.3. Sample Size

The relationship between self-care and HRQoL was analyzed using structural equation modeling (SEM). Since this was a secondary analysis, post hoc analysis was conducted to assess the power and minimum sample size required to estimate the root mean square error of approximation (RMSEA), the main index used to evaluate the SEM fit. With a sample of 521 individuals, the power to reject the null hypothesis of RMSEA was >0.90. Additionally, given a power of 0.80, an alpha of 0.05 (two-tailed), and 248 degrees of freedom for the main SEM, the minimum sample size required to estimate the RMSEA was 99 [30].

## 3. Instruments

### 3.1. Sociodemographic Variables

Our research group created an ad hoc questionnaire to collect the patients’ characteristics including age, sex, marital status, employment, education, income received, type of stoma, whether the stoma was permanent, stoma-related complications during hospitalization, time since stoma placement, and presence of co-morbid conditions.

### 3.2. Stoma-Specific Quality of Life Questionnaire (Stoma QoL)

The Stoma QoL is a 20-item questionnaire used to assess QoL in patients with ostomy [31]. The items are rated on a 4-point Likert scale and discuss the frequency of fear, worry, difficulty, and shame that individuals with ostomy feel because of their condition in their daily lives. The score ranges vary between 20 and 80; higher scores indicate higher HRQoL. In our sample, the reliability computed using the omega coefficient (ω) was good (0.98).

### 3.3. Ostomy Self-Care Index (OSCI)

The OSCI is a 32-item questionnaire developed to measure self-care practices in patients with ostomy [29]. Each item is rated on a 5-point Likert scale from 1 (never/rarely) to 5 (always/daily). The OSCI captures various dimensions of self-care, including self-care maintenance (i.e., daily routine behaviors), self-care monitoring (i.e., stoma and peristomal skin inspection), self-care management (i.e., problem recognition and related response behaviors), and self-care self-efficacy (i.e., confidence in the ability to engage effectively in self-care). Higher scores on each subscale (in a range of 0–100) indicate higher levels of self-care. In this study, only a small proportion of the patients completed the management subscale (n = 84, 16.1%) because of the absence of ostomy-related issues. Therefore, this subscale was not used in the main model. The reliability measures of the three subscales were satisfactory in our data (self-care maintenance: ω = 0.97; self-care monitoring: ω = 0.95; self-care self-efficacy: ω = 0.96).

### 3.4. Ethical Considerations

The Institutional Review Board Committee granted permission to conduct this study (Ref. number 19159/18, ID2075). This study adhered to the principles stated in the Helsinki Declaration. Patients granted written, informed permission after being informed of the study aims. All data collected were stored securely and utilized only for the objective of this scientific investigation.

## 4. Data Analysis

The relationship between self-care and HRQoL was investigated within the SEM framework. Latent factors were specified using parcels; this strategy was adopted to reduce the number of parameter estimates and the likelihood of correlated errors. The parceling approach also had further advantages, such as maximization of reliability and true-score variance of the model.

The parcels were created with the balancing approach, considering the unidimensional structure of the constructs of interest. Briefly, the items were summed and allocated on the bases of the total-item corrected correlations; the latent constructs were based on pairs of items, except for the QoL, which included 20 items and was based on quadruplets of items.

An SEM is characterized by a measurement model and a structural model. The measurement model was examined with confirmatory factor analyses, in which each model was specified with the latent construct and its related parcel indicators. The validity of the models was confirmed with the following fit indices: comparative fit index (CFI), Tucker–Lewis index (TLI), root mean square error of approximation (RMSEA), and standardized root mean square residual of approximation (SRMR). Indication of supportive fit was obtained with CFI and TLI values of 0.95 or greater, RMSEA values of 0.008 or lower, and SRMRs of 0.008 or lower [32,33]. X^2^ statistics were also reported but not used to judge model fit because of their high sensitivity to sample size [34]. The internal consistency reliability of each model was judged using the omega coefficient; values equal to or higher than 0.70 were considered supportive.

After establishing the validity of the measurement model, the structural model was tested by specifying the structural coefficients between the latent variables. Specifically, the analysis tested a model in which the two self-care constructs (i.e., maintenance and monitoring) influenced HRQoL. This model was adjusted for several covariates according to the literature (i.e., age, marital status, occupation, living conditions, education, and underlying oncological disease) [12,16,17,35]. The model fit of the structural model was judged according to the same fit indices.

The main analysis was complemented by examining the descriptive statistics of the sample population. The normality assumption was verified by inspecting the skewness and kurtosis values of each parcel, for which values above |2| indicate a non-negligible departure from a normal distribution. Missing value analysis was also conducted for each parcel.

## 5. Results

### 5.1. Sociodemographic Characteristics of the Sample

Briefly, the sample population (n = 521) had a mean age of 68.65 years (SD = 12.45) and was mostly male (63.9%), married or partnered (70%), and unoccupied or retired (67.5%), while a minority lived alone (17.6%). Almost 40% of the participants had a colostomy, and 82.8% had an ostomy placed for oncological reasons (Table 1).

### 5.2. Preliminary Analysis

The skewness and kurtosis values of the parcels were all within the |2| range, indicating no violation of the normality assumption. Missing data were not present.

### 5.3. Measurement Model

The fit indices of the three latent constructs are summarized in Table 2. The standardized factor loadings were all high (>0.80) and significant. The internal consistency was also high, with omega coefficients ranging from 0.96 to 0.98 (Table 2).

### 5.4. Structural Model

**Figure 1:**
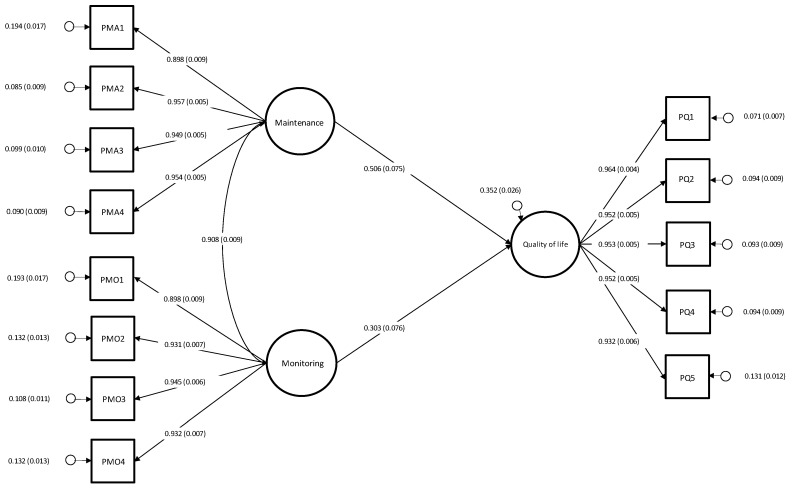
Structural equation model of the study (n = 521).

The hypothesized model (Figure 1) yielded the following fit indices: χ^2^ (146, N = 521) = 511.731, *p* ≤ 0.001, CFI = 0.97, TLI = 0.96, RMSEA = 0.069 (90% CI, 0.063–0.076), *p* ≤ 0.001, and SRMR = 0.038. Only two covariates reached statistical significance (i.e., age: β = 0.12, *p* < 0.001, and presence of underlying oncological disease: β = −0.06, *p* = 0.030); therefore, to avoid overcontrol, the parameters of the unsignificant covariates were fixed to zero [36]. The chi square difference test indicated that constraining these paths to zero did not worsen model fit: ∆ χ^2^(5) = 4.716, *p* = 0.452. The final model exhibited acceptable fit indices: χ^2^ (151, N = 521) = 516.447, *p* ≤ 0.001, CFI = 0.97, TLI = 0.96, RMSEA = 0.068 (90% CI, 0.062–0.075), *p* < 0.001, and SRMR = 0.038. Self-care maintenance and self-care monitoring had a positive effect on ostomy-related QoL (maintenance: β = 0.506, *p* < 0.001; monitoring: β = 0.303, *p* < 0.001).

## 6. Discussion

This study investigated the association between self-care and HRQoL in patients with ostomies. To the best of our knowledge, this is the first study to explore this relationship among individuals with ostomy through robust statistical analysis.

Our results indicate that self-care maintenance and monitoring had a direct positive influence on HRQoL, confirming the relationship between self-care behaviors and HRQoL. Our research indicates that self-care is a predictor of HRQoL and plays a critical role in improving the health and well-being of patients with ostomies. This is particularly relevant because the previous literature examining the relationship between HRQoL and self-care in patients with ostomies used measures that underrepresented the construct of self-care [12,19,20,21,22]. While earlier studies recognized a positive association between HRQoL and self-care, our research strengthens this link by utilizing a more comprehensive measure of self-care and employing a robust statistical method. The relationship between self-care and HRQoL is well-documented in other chronic disorders [24,25,26,27,37]. The findings of our study, therefore, not only align with previous literature on chronic illnesses but also extend this body of knowledge by focusing on the unique needs of patients with ostomies.

Our study also found a positive relationship between age and HRQoL. This is consistent with previous studies conducted on individuals living with a stoma [18,38]. Several reasons may explain this association. First, our sample was predominantly composed of elderly individuals who carried a permanent stoma, which suggests that they have had more time to adjust to the stoma-related lifestyle changes than their younger counterparts. Second, those who have a long-term stoma tend to gain stronger confidence and competence in stoma care than those with less experience with the device. All these factors may have ultimately enhanced the HRQoL in these individuals [39].

Moreover, our study sustains the negative correlation between underlying cancer diagnoses and HRQoL in individuals with ostomy. Previous findings show that, among patients with ostomies, individuals with cancer, particularly colorectal cancer, generally reported lower HRQoL compared to individuals with ostomies but without cancer. This reduction in quality of life was often attributed to several interrelated factors: the psychological burden of a cancer diagnosis, the physical demands of ostomy management, and the social stigma associated with both conditions [40,41]. The stress of coping with cancer exacerbates these psychological challenges, contributing to feelings of despair and an overall reduction in well-being [4]. In contrast, Jansen et al. [42] revealed that, with the exception of mental health issues, patients with ostomies with cancer reported a higher generic HRQoL than patients with ostomies without cancer. Other research did not find any differences in HRQoL among patients with ostomies due to inflammatory diseases, colon cancer, intestinal disorders, or other reasons [43]. Finally, in contrast with previous research, our study stressed that variables such as marital status, occupation, living conditions, and education level did not have a significant impact on the HRQoL of patients with ostomy [12,16,17,35]. Given the heterogeneity of results across studies, future research on variables that influence HRQoL would be desirable.

The results of this research carry significant theoretical implications, as they lend support to self-care theories by validating the connection with HRQoL within the ostomy population. This supports the importance of assessing self-care among individuals with ostomies, improving interventions to support self-care practices, and monitoring self-care outcomes [44,45]. A scoping review by Riegel et al. [46] highlights that self-care interventions for patients with chronic conditions should generally include several key components including patient education, behavioral support, regular feedback mechanisms, and the use of digital health tools like apps and telemonitoring. Nurses play a crucial role in this process by collaborating to create personalized care plans, tailoring chronic disease management strategies to meet individual patient needs, and facilitating the monitoring and adherence to self-care practices [47].

The study findings also have clinical and research implications. Previous studies that developed strategies to implement self-care practices for patients with ostomy with effects on HRQoL lacked a theoretical underpinning and did not focus on casual contributors to outcomes [45]. Our findings provide a clearer framework for future research, which could focus on developing targeted, theory-based interventions aimed at improving self-care in this population. Such interventions should incorporate robust self-care models and use validated instruments to measure self-care behaviors, ensuring that healthcare providers can accurately assess individual skills, deliver focused health education, and effectively monitor outcomes [28,48].

This study carries several limitations. First, the cross-sectional design impedes a comprehensive analysis of the causal relationships among the variables. Consequently, longitudinal studies should be conducted to further confirm the nature of such relationships. Secondly, our study included a convenience sample drawn from Italy, which may limit the generalizability of the findings to the broader ostomy population. Finally, no moderators or mediators were included in the SEM. This approach would have been useful to improve the understanding of the mechanisms involved in the relationships between the variables and how external factors may influence outcomes. Despite these limitations, our study offers notable strengths. First, we included a large sample size from several healthcare organizations, which led to greater variability in the data and robust statistical analysis. Second, this study employed an SEM framework to analyze latent variables, thereby enhancing the validity of the findings. By accounting for the measurement error associated with the indicators, our analysis provides a more accurate estimation of the relationship between self-care and HRQoL.

## 7. Conclusions

This study provides evidence supporting the association between HRQoL and self-care, indicating that insufficient self-care practices lead to a low HRQoL. Consequently, to enhance the HRQoL of patients with ostomy, interventions must be developed to improve self-care behaviors. Additional research is warranted to identify the variables that may act as potential moderators and/or mediators in the relationship between self-care and HRQoL.

## Figures and Tables

**Table 1:** Descriptive statistics of the participants (n = 521).

Sample Characteristics	n (%)
Gender (female), *n*, *%*	189 (36.1)
Age (years), *m*, *SD*	68.65 (12.45)
Civil status (married/partnered), *n*, *%*	366 (70)
Occupation (unoccupied/retired), *n*, *%*	353 (67.5)
Education (<9 y), *n*, *%*	266 (50)
Perceived income (adequate), *n*, *%*	427 (81.6)
Live alone, *n*, *%*	92 (17.6)
Type of stoma, *n %*	
Colostomy	203 (38.8)
Ileostomy	153 (29.3)
Urostomy	158 (30.2)
Permanent stoma, *n*, %	379 (72.5)
Stoma-related illness, *n*, % (Oncologic)	433 (82.8)
Stoma-related complications during hospital stay (no), *n*, *%*	411 (78.6)
Time since stoma placement (months), *m*, *SD*	40.6 (69.47)
Comorbidities, *n*, *%*	242 (46.3)

Note: SD, standard deviation.

**Table 2:** Fit indices and reliability coefficients of the CFA models (n = 521).

Model	χ^2^ (df)	P (χ^2^)	RMSEA *	RMSEA * (90%CI)	*p* (RMSEA * < 0.05)	CFI ^§^	TLI ^†^	SRMR ^||^	AVE ^¶^	CR ^‡^
Self-care maintenance	5.155 (1)	0.023	0.089	0.026–0.171	0.130	0.998	0.989	0.004	0.87	0.96
Self-care monitoring	4.811 (2)	0.090	0.052	0.000–0.113	0.383	0.998	0.995	0.005	0.86	0.96
HRQoL	10.411 (4)	0.034	0.055	0.014–0.098	0.352	0.998	0.996	0.003	0.89	0.98

* RMSEA = root mean square error of approximation, ^§^ CFI = comparative fit index, ^†^ TLI = Tucker–Lewis index, ^||^ SRMR = standardized root mean square residual, ^¶^ AVE = average variance extracted, ^‡^ CR = composite reliability.

## Data Availability

The dataset used for this study is available upon reasonable request from the authors.

## Public Involvement Statement:

No public involvement in any aspect of this research.

## Guidelines and Standards Statement:

This manuscript was drafted against the STROBE (Strengthening the reporting of observational studies in epidemiology) for cross-sectional studies. A complete list of reporting guidelines can be accessed via the equator network: https://www.equator-network.org/.
